# Is oestrogen receptor-negative/progesterone receptor-positive (ER−/PR+) a real pathological entity?

**DOI:** 10.3332/ecancer.2021.1278

**Published:** 2021-08-24

**Authors:** Adedayo A Onitilo, Jessica Engel, Adedayo O Joseph, Ya-Huei Li

**Affiliations:** 1 Department of Oncology, Marshfield Clinic Health System-Weston Center, 3501 Cranberry Blvd, Weston, WI 54476, USA; 2 Cancer Care and Research Center, Marshfield Clinic Research Institute, Marshfield, WI 54449, USA; 3 NSIA-LUTH Cancer Treatment Center, Lagos University Teaching Hospital, Ishaga Rd, Idi-Araba 102215, Lagos, Nigeria

**Keywords:** breast cancer, borderline oestrogen-receptor, progesterone-receptor-positive, recurrence score, multigene assay

## Abstract

**Background:**

The existence of oestrogen receptor-negative (ER−)/progesterone receptor-positive (PR+) breast cancer continues to be an area of controversy amongst oncologists and pathologists.

**Methods:**

To re-evaluate breast cancers originally classified as ER−/PR+ via Oncotype DX® assay and compare molecular phenotype with Recurrence Score® (RS) result, clinicopathologic features and clinical outcomes were retrospectively obtained from electronic health records between January 1998 and June 2005. Archived formalin-fixed, paraffin-embedded (FFPE) tumour specimens were tested for the expression of ER, PR and human-epidermal-growth-factor-2. The number of positive ER−/PR+ samples confirmed by transcriptional analysis was the primary outcome of interest with event-free and overall survival as secondary outcomes. Biopsies from 26 patients underwent Oncotype DX testing and analysis.

**Results:**

Approximately 60% were middle-aged (40–50 years old) women, and 84.6% had invasive ductal carcinoma. Based on the Oncotype DX assay, approximately 65% (*N* = 17) had ER+/PR+ status; 23% (N = 6) had ER−/PR− status; and 12% had a single hormone positive receptor (1 ER–/PR+, 2 ER+/PR–) status. Almost one-quarter of patients were stratified into the low-RS (<18) or intermediate-RS (18–30) results, and half of the patients had a high-RS (>30) result.

**Conclusion:**

Our findings suggest the ER−/PR+ subtype is not a reproducible entity and emphasises the value of retesting this subtype via molecular methods for appropriate treatment selection and patient outcomes. Multigene assay analysis may serve as a second-line or confirming tool for clinical determination of ER/PR phenotype in breast cancer patients for targeted therapies.

## Introduction

Breast cancer is not one disease but comprises a heterogeneous disease group of a growing number of biological subtypes that have diverse natural histories and responsiveness to treatments. Determination of appropriate breast cancer treatments and prognostic outcomes are dependent on the accurate histologic classification and measurement of two main biomarker groups: hormone receptors (HRs; including oestrogen (ER) and progesterone receptors (PR)) and human epidermal growth factor 2 (HER2) [1]. Of the HRs, ER expression is predictive in identifying tumours that will respond to endocrine therapy, whereas PR expression is prognostic with patients exhibiting PR− breast cancers having poor outcomes [2–4]. Endocrine therapy is considered the most effective treatment to downregulate ER-related cancer cell functions with relatively low toxicity compared to cytotoxic chemotherapy in patients with ER+ breast cancer [1]. On the other hand, cytotoxic chemotherapy is indicated for patients with low expression of HRs because of their documented insensitivity to endocrine therapy [1]. Although at higher risk for chemotherapy-related adverse events, women with HR−/HER2+ or HR−/HER2− triple negative breast cancers gain more survival benefit from systemic chemotherapy [1]. As such, accurate definition of biomarker status, including HR expression, is critical for breast cancer diagnosis and treatment.

Immunohistochemistry (IHC) is a currently used technique to measure the level of ER and PR biomarker expression in breast cancer tissues and evaluate cancer responsiveness to endocrine therapy. Development and improved sensitivity and specificity of antibodies [5] as well as the establishment of lower cut-offs of >1% have improved IHC assay performance and contributed to the identification of ER+/PR+, ER+/PR− and ER−/PR− breast cancer subtypes [6]. However, the possible existence of an ER−/PR+ breast cancer remains in dispute. Over time, fewer ER−/PR+ breast cancers have been reported in Surveillance, Epidemiology, and End Results cancer registry data from 4.5% in the early 1990s to 1% in recent years [7], and recent studies of ER−/PR+ breast cancer analysis by repeat IHC using updated methods produced many reclassifications and a significant reduction in the number of ER−/PR+ cases [8–10]. Thus, whether the ER−/PR+ subtype is an artefact or bonafide breast cancer entity that requires specific treatment regimens is still controversial.

The goal of the study was to evaluate whether ER−/PR+ HR status in breast cancer is a true pathological entity using the molecular assay Oncotype DX results as a comparison to our institutional results for receptor status. The Oncotype DX assay is a reverse-transcription polymerase chain reaction (RT-PCR)-based test that measures the expression levels of ER, PR and HER2 mRNA as well as 21 genes associated with cancer progression and patient outcomes. Individual ER, PR and HER2 expression scores are provided as well as a Recurrence Score® (RS^TM^) result generated from a validated algorithm that compares the expression levels of 16 cancer-related genes compared to the expression of 5 housekeeping control reference genes to predict the risk of disease recurrence within 10 years after treatment [11, 12]. The Oncotype DX assay has been incorporated into standard-of-care recommendations for breast cancer treatment (National Comprehensive Cancer Network guidelines) and has documented predictive value in several large breast cancer clinical trials including National Surgical Adjuvant Breast and Bowel Project (NSABP) trials, the Arimidex, Tamoxifen, Alone or in Combination trial, the Trial Assigning Individualized Options for Treatment (TAILORx) and others [11, 13–18].

By re-examining the phenotype and associated patient outcomes of breast cancer biopsies previously designated as ER−/PR+ with updated techniques, our results suggest that ER−/PR+ tumours are not a reproducible pathological entity, though small sample size precludes definitive conclusions regarding the existence or non-existence of this subtype. Follow-up transcriptional analysis of tumour samples classified as ER−/PR+ by IHC staining may assist oncologists in selecting the appropriate treatment regimen for patients diagnosed with breast cancer.

## Methods

### Study design and regulatory statement

The Institutional Review Board at the Marshfield Clinic Research Institute (IRB-18-306) approved this retrospective analysis of clinical specimens and patient records.

### Patient selection

The records of all adult (≥18 years of age) female patients with a diagnosis of invasive primary breast cancer and designated ER−/PR+ IHC classification between 1 January 1998 and 30 June 2005 were reviewed for analysis. These dates were selected according to institutional adoption of the 5% staining cutoff for HR positive status according to the American Society of Clinical Oncology/College of American Pathologists (ASCO-CAP) guidelines at the time. Patient characteristics, stage and grade of cancer at diagnosis, nodal disease status, tumour size, treatment options, tumour location and dates of recurrence and patient deaths were retrospectively captured. Overall survival was defined as the period between breast cancer diagnosis and death event or censored observation. Event-free survival was defined as the time between breast cancer diagnosis and recurrence/death/censored observation. Corresponding archived acid-FFPE tumour specimens were obtained from and sent to Genomic Health, Inc. for Oncotype DX analysis.

### Oncotype DX gene expression analysis

The Oncotype DX assay (Genomic Health, Inc., Redwood City, CA) is a quantitative RT-PCR (qRT-PCR) assay used to measure the expression of 21 genes (5 reference genes and 16 cancer-related genes) in FFPE breast cancer tissues. We used the Oncotype DX assay to evaluate ER/PR receptor status at the RNA level and calculate the breast cancer RS result to evaluate the association between HR status and clinical outcomes in our cohort. The RS result is derived within the Oncotype DX assay via a validated algorithm that compares the expression of the 16 cancer-related genes and five control reference genes to clinical outcomes of breast cancer ~ 6,000 patients in the NSABP and TAILORx clinical trials [11, 12, 14–16, 18]. The RS result is stratified into three risk categories for poor patient outcomes: low (<18), intermediate (18–30) and high (>30) [11, 12, 14]. This result was compared to the expression levels of individual HER2, ER and PR genes, and patient samples phenotyped according to pre-defined gene expression levels for positive, equivocal or negative IHC staining correlation [19–21]. Expression level ≥ 11.5 units of HER-2 is categorised as high/positive; 10.7–11.4 units: equivocal/indeterminate; and <10.7 units: negative [20]. Similarly, expression of ER and PR is categorised as high/positive ≥ 6.5 units and ≥5.5 units, respectively [21].

### Statistical analysis

Descriptive analyses of means, standard deviations, frequency counts and percentage of the subpopulations were conducted. Scatter charts comparing RS result and event-free/overall survival were plotted for assessment of correlation. All statistical analyses were generated using SAS software 9.4 (SAS Institute, INC., Cary, NC, USA).

## Results

We identified 28 patient samples originally classified as ER−/PR+ breast cancer based on IHC results. Biopsies from two patients were excluded due to insufficient tumour RNA while the remaining biopsy samples from 26 patients were analysed via Oncotype DX assay.

Patient characteristics, tumour characteristics, patient outcomes, treatment options and Oncotype DX assay results are presented in Table 1. The majority of patients (57.7%) were between the ages of 40 and 50, and 84.6% had invasive ductal carcinoma. Based on primary IHC results, approximately 40% of patients did not receive endocrine therapy at the time of diagnosis. Most patients (>90%) had surgery and systematic chemotherapy (81%). Two of the 26 patients did not have any type of surgery. Twenty-three (88%) patients had a stage I or II tumour at the time of diagnosis and approximately half of them had radiotherapy (53.9%) and endocrine therapy (61.5%). Approximately half of the patients had node-positive, grade III and small (≤2 cm^2^) breast cancers. Twelve patients (46.2%) died during the study period and 14 patients (53.9%) experienced recurrence or death overall.

Oncotype DX analyses revealed that 17 of the 26 patients (65.38%) were ER+/PR+, 2 (7.69%) were ER+/PR− and 6 (23.08%) were ER−/PR−. Only one (3.85%) patient out of the entire cohort was confirmed to have an ER−/PR+ breast tumour. Four patients (15.38%) were HER2+, and two patients (7.69%) had equivocal HER2 expression. The mean RS result was 35.9 ± 21.1. Approximately one-quarter of the patients had a low RS result and half of the patients had a high RS result.

Further analysis of patient records for treatment history indicated a discrepancy in treatment selection according to the revised molecular phenotyping of corresponding clinical samples. For women with Oncotype Dx-confirmed ER+ status, two (11%) women had endocrine therapy alone, four (21%) had chemotherapy alone and eleven (58%) had both endocrine and chemotherapy. Conversely, two-thirds of patients (4 out of 6) with confirmed ER−/PR− by Oncotype DX had triple-negative breast cancers. Of these patients, one (25%) had endocrine therapy only, one (25%) had both endocrine and chemotherapy and two (50%) had chemotherapy alone. Out of the entire cohort, only one patient (H1) was identified as ER−/PR+ by the Oncotype DX assay. This 56-year-old patient had a history of multiple cancer types with metastases prior to her breast cancer diagnosis. Therefore, it is difficult to evaluate whether treatment selection was adequate for her cancer regardless of additional molecular testing.

**Figure 1.**
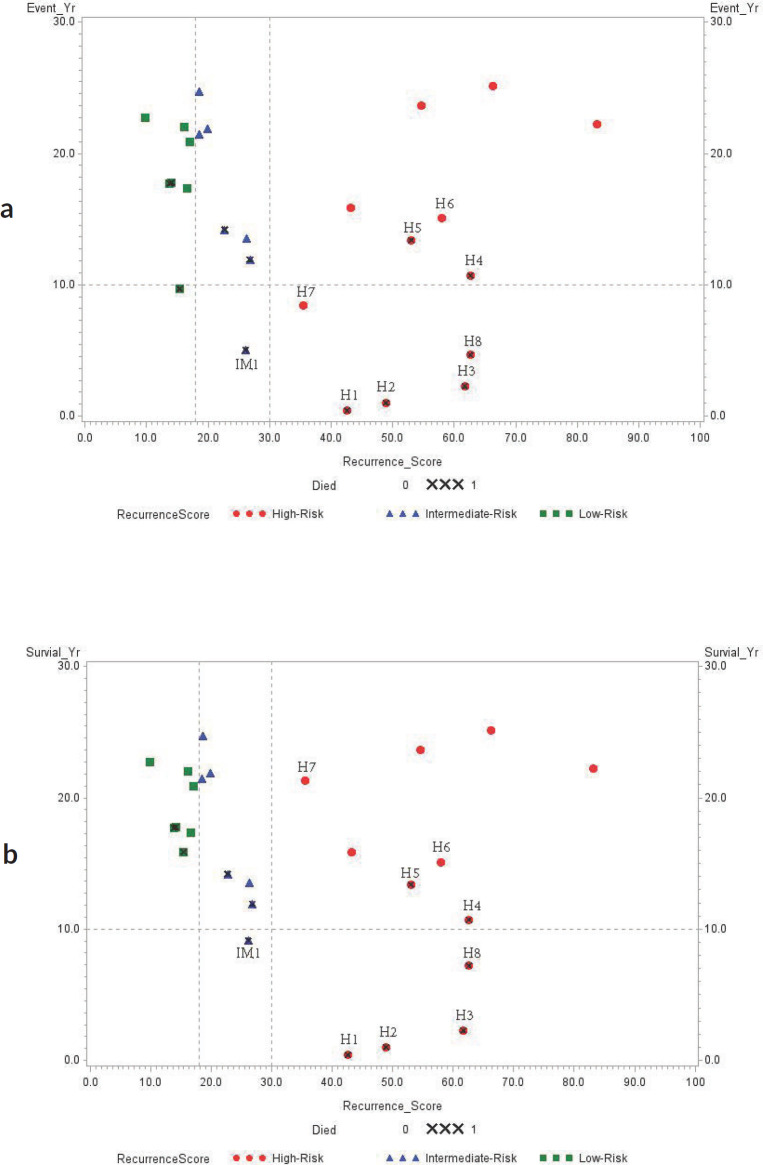
(a): Event-free survival (year); (b): Overall survival (year). Association between RS result and years of survival amongst study subjects. RS result (0–100) is categorised by the risk of recurrence using different colour symbols: green square (RS result < 18, low-risk), blue triangle (RS result 18–30, intermediate-risk) and red dot (RS result > 30, high-risk). ‘X’ symbol indicates deceased patients.

Spearman correlation analysis of the 12 deceased patients showed that increasing RS result was positively associated with decreased overall survival (r^2^ = −0.6669, *p* = 0.0178). Scatter plots with cutoff RS results at 18 (low risk for poor outcome) and 30 (high risk for poor outcome) compared to years of event-free and overall survival are presented in Figure 1. Four patients with high RS result had triple-negative (ER−/PR−/HER2−) breast cancers: H3 (RS result 62), H4 (RS result 63), H5 (RS result 53) and H6 (RS result 58) while two patients with high RS results (H7: RS result 36 and H8: RS result 63) had HER2+/ER+/PR+ breast cancers.

## Discussion

Molecular re-evaluation of biopsies from 26 patients with IHC classified ER−/PR+ status using the Oncotype DX 21-gene expression assay revealed that most patients classified as ER−/PR+ were actually ER+ and may have benefited from endocrine therapy. Only one patient was confirmed to have ER−/PR+ status which suggests that ER−/PR+ breast cancers may not be a bonafide pathological entity. Retesting of breast cancers initially classified as ER−/PR+ by IHC with transcriptional-based methods like the Oncotype DX assay is essential for oncologists to accurately select treatment, reduce patient risks from over- and under-treatments and to improve patient outcomes.

Some researchers believe ER−/PR+ is a distinct breast cancer subtype that occurs in younger premenopausal women with poorly differentiated tumours and is rarely of classical lobular type [22–24]. They report an association between ER−/PR+ status with biomarkers of poor prognosis, such as positive p53 and basal cytokeratins as well as reduced E-cadherin expression, and emphasise that women with ER−/PR+ breast cancer tend to have unfavourable outcomes compared to those with ER+/PR+ status [3, 25, 26]. Furthermore, a variant ER that lacks exon 5 of the hormone-binding domain and can stimulate the ER-responsive PR expression has been discovered and is cited as one potential mechanism to explain the existence of an ER−/PR+ breast cancer [27]. Such a mechanism would suggest that women with this receptor could still induce PR expression through the ER pathway and produce tumours that are sensitive toward endocrine therapy.

Conversely, others claim that ER−/PR+ is a technological artefact that cannot be reproduced and suggest reassessment when an ER−/PR+ IHC result occurs [8, 10, 28–30]. Analysis of IHC techniques for HR expression suggests that less than 6-hours staining or a prolonged process in tissue fixation and varied immune-reactivity for ER and PR can hinder the ability of IHC antibodies to detect weak ER+ staining [31–33]. Furthermore, high levels of oestrogen in young women can saturate the ER thus preventing ligand binding to the ER of the lesion [2, 34].

Amongst the 26 cases analysed, 65% (*N* = 17) were determined to have ER+/PR+ expression, 23% (*N* = 6) had ER−/PR− expression and 12% had single HR expression (2 ER+/PR− and 1 ER−/PR+). If this profile of gene expression had been provided in the 1990s, 19 of the patients (73%) would have qualified for and potentially benefitted from endocrine therapy without the increased risk of adverse events associated with chemotherapy. Appropriate endocrine therapy was largely withheld from this borderline ER+ group, most of whom received chemotherapy, which is not very effective in ER+ breast cancers. Such misclassification leads to inadequate treatment selection that compromise treatment effects and expose patients to unnecessary risks of side effects from chemotherapy. In contrast, 50% of patients originally classified as ER–/PR+ were found to have triple-negative breast cancer; of these patients, one received inaccurate treatment and one had endocrine therapy added to manage disease, emphasising the necessity of accuracy in the identification of ER receptor status to guide the appropriate clinical treatment.

Only one of the 26 breast cancer patients was confirmed to have ER−/PR+ (ER: 5.5 units, PR: 6.1 units, HER2: 7.3 units) status by the Oncotype DX assay in our study. These Oncotype DX results support the idea that ER−/PR+ breast cancers are not a bonafide pathological entity. Other studies that conducted repeat IHC testing for ER−/PR+ breast cancers reclassified the samples 45%–85% of the time, of which, the most frequent change was to ER+ status [8, 10, 28]. Thus, it seems optimal that some type of repeat testing (IHC or molecular) for ER−/PR+ breast cancers is required for timely treatment selection, as recommended in the 2010 ASCO-CAP guidelines [6]. One considerable advantage of the Oncotype DX assay over repeat IHC or another molecular approach is that Oncotype DX provides the RS result that identifies high-risk patients who will benefit from chemotherapy amongst the biologically diverse group of low ER expressing breast cancers.

Stratification of our study patients by RS result confirmed that the RS cutoff correlates with risk of recurrence or mortality, as 14 of the 26 cases had <30 RS result (low- or intermediate-risk) and lived ≥ 10 years after diagnosis. On the other hand, 20% of our no-metastatic cancer patients with a >30 RS result lived less than 10 years. Iwamoto *et al* found that a majority of borderline ER+ breast cancers based on IHC had a basal-like molecular expression profile and therefore would likely benefit from the use of both adjuvant endocrine therapy and chemotherapy [35].

Our study should be interpreted conservatively based on the following limitations. First, the sample size of this study was relatively small and with limited power. Second, the study was a retrospective review of patient documents and testing of corresponding paraffin-embedded tissues obtained in the late 1990s. Missing information and information bias is inherent to the retrospective approach. Furthermore, due to changes in treatment strategies over time, alternative treatment options might now be suggested by additional tests that might alter the correlation between RS result, treatment selection and survival. Third, our breast cancer specimens were processed in an acid-formalin fixative, which is known to have variable effects on mRNA stability. However, the Oncotype DX assay uses expression levels of the five reference control genes as a measure of overall mRNA quality. Finally, our study compares somewhat subjective IHC interpretation results to Oncotype DX molecular results as well as to the Oncotype DX cutoffs for positive expression of HER2, ER and PR which were established based on validation against IHC results from hundreds of breast cancer specimens from clinical trials that may not match the demographic and socioeconomic characteristics of our sample population [20, 21]. However, given the large number of breast cancer samples used to develop and validate the Oncotype DX assay as well as studies that have established an excellent correlation between ER, PR and HER2 mRNA and protein levels [11, 12, 15–21], ensure that our comparisons are reasonable and accurate.

Despite such limitations, our findings seem comparable to the small number of prior studies that have examined molecular characteristics of ER−/PR+ breast cancers [8, 10, 28]. This study adds new insight on borderline (<5%) ER-receptor expression of IHC and rises pathologist and oncologist awareness regarding the importance of re-testing clinical specimens of patients with borderline ER-receptor expression, which echoes the urge of ASCO-CAP panel to pay more attention to the true benefit of endocrine therapy in patients with 1%–10% borderline ER−positive cancer [36].

## Conclusions

Though our study has limited ability to make broad conclusions with respect to appropriate treatment options for patients with borderline ER+ expression and existence of the ER−/PR+ breast cancer subtype, it emphasises the need for retesting patients classified with ER−/PR+ breast cancer and suggests the clinical rarity of this pathological subtype. Our findings highlight the potential clinical utility of multigene molecular testing to support clinical decision-making in the diagnosis and treatment of patients with breast cancer and recommend molecular confirmation of indeterminate HR expression results by standard IHC.

## List of abbreviations

ASCO-CAP, American Society of Clinical Oncology/College of American Pathologists;

ER, Oestrogen receptor;

FFPE, Formalin-fixed, paraffin-embedded;

HER2, Human epidermal growth factor 2;

HR, Hormone receptor; IHC, Immunohistochemistry;

NSABP, National Surgical Adjuvant Breast and Bowel Project;

PR, Progesterone receptor;

qRT-PCR, Quantitative reverse-transcription-polymerase-chain-reaction;

RS, Recurrence score;

RT-PCR, Reverse-transcription polymerase chain reaction;

TAILORx, Trial Assigning Individualized Options for Treatment.

## Funding information

Genomic Health, Inc. absorbed the cost of gene expression profiling of the tissue specimens with Oncotype DX panel.

## Conflicts of interest disclosure

The authors have no personal or financial conflicts of interest to disclose.

## Authors’ contributions

Concept and design: AAO, JE, AOJ, YHL. Data analysis: AAO, JE, AOJ, YHL. Manuscript writing, proofreading, final approval: AAO, JE, AOJ, YHL.

## Figures and Tables

**Table 1.** Patient characteristics, tumour characteristics, treatment history, outcomes and Oncotype DX assay results (*N* = 26).

Patient and tumour characteristics		
Age (years)	Mean (SD; range)	53.27 (14.63, 32–86)
	<40 years	5 (19.23)
	41–50 years	10 (38.46)
	51–60 years	4 (15.38)
	61–70 years	3 (11.54)
	>70 years	4 (15.38)
Histology	Ductal in situ carcinoma	1 (3.85)
	Invasive ductal carcinoma	22 (84.62)
	Invasive lobular carcinoma	2 (7.69)
	Missing	1 (3.85)
Nodal status	Negative	12 (46.15)
	1–3 positive nodes	9 (34.62)
	>3 positive nodes	3 (11.54)
	Missing	2 (7.69)
Tumour grade	I	4 (15.38)
	II	7 (28.00)
	III	14 (56.00)
	Missing	1 (3.85)
Tumour stage	I	10 (38.46)
	II	13 (50.00)
	III	1 (3.85)
	IV	2 (7.69)
Tumour size (cm^2^)	<1.0	5 (19.23)
	1.0–1.5	8 (30.77)
	1.6–2.0	5 (19.23)
	2.1–4.0	4 (15.38)
	>4.0	3 (11.54)
	Missing	1 (3.85)
Treatment		
	Surgerya	24 (92.31)
	Radiotherapy	14 (53.85)
	Endocrine therapyb	16 (61.54)
	Chemotherapy	21 (80.77)
**Patient outcomes**		
Overall survival (years)	Mean (SD)	15.75 (7.22)
	Deaths	12 (46.15)
Event-free survival (years)	Mean (SD)	14.76 (7.63)
	Event (recurrence/deaths)	14 (53.85)
**Oncotype DX testing**		
ER	Positive	19 (73.08)
PR	Positive	18 (69.23)
	ER+/PR+	17 (65.38)
	ER+/PR−	2 (7.69)
	ER−/PR+	1 (3.85)
	ER−/PR-	6 (23.08)
HER2	Negative	20 (76.92)
	Equivocal	2 (7.69)
	Positive	4 (15.38)
RS	Mean (SD)	35.92 (21.10)
	Low risk (<18)	7 (26.92)
	Intermediate risk (18–30)	7 (26.92)
	High risk (≥31)	12 (46.15)

^a^Two no-surgery patients had a metastatic disease

^b^Two of the four were reclassified as triple-negative and had endocrine therapy

Abbreviations: ER, Oestrogen receptor; PR, Progesterone receptor; HER2, Human epidermal growth factor 2

## References

[1] Waks AG, Winer EP (2019). Breast cancer treatment: a review. J Am Med Assoc.

[2] Kiani J, Khan A, Khawar H (2006). Estrogen receptor alpha-negative and progesterone receptor-positive breast cancer: lab error or real entity?. Pathol Oncol Res.

[3] Itoh M, Iwamoto T, Matsuoka J (2014). Estrogen receptor (ER) mRNA expression and molecular subtype distribution in ER-negative/progesterone receptor-positive breast cancers. Breast Cancer Res Treat.

[4] Wu N, Fu F, Chen L (2020). Single hormone receptor-positive breast cancer patients experienced poor survival outcomes: a systematic review and meta-analysis. Clin Transl Oncol.

[5] Saccani Jotti G, Johnston S, Salter J (1994). Comparison of new immunohistochemical assay for oestrogen receptor in paraffin wax embedded breast carcinoma tissue with quantitative enzyme immunoassay. J Clin Pathol.

[6] Hammond M, Hayes D, Dowsett M (2010). American society of clinical oncology/college of American pathologists guideline recommendations for immunohistochemical testing of estrogen and progesterone receptors in breast cancer (unabridged version). Arch Pathol Lab Med.

[7] Li Y, Yang D, Yin X (2020). Clinicopathological characteristics and breast cancer-specific survival of patients with single hormone receptor-positive breast cancer. JAMA Netw Open.

[8] Maleki Z, Shariat S, Mokri M (2012). ER-negative/PR-positive breast carcinomas or technical artifacts in immunohistochemistry?. Arch Iran Med.

[9] Ahmed SS, Thike AA, Zhang K (2017). Clinicopathological characteristics of oestrogen receptor negative, progesterone receptor positive breast cancers: re-evaluating subsets within this group. J Clin Pathol.

[10] Foley N, Coll J, Lowery A (2018). Re-appraisal of estrogen receptor negative/progesterone receptor positive (ER-/PR+) breast cancer phenotype: true subtype or technical artefact?. Pathol Oncol Res.

[11] Paik S, Shak S, Tang G (2004). A multigene assay to predict recurrence of tamoxifen-treated, node-negative breast cancer. N Engl J Med.

[12] Sparano JA, Paik S (2008). Development of the 21-gene assay and its application in clinical practice and clinical trials. J Clin Oncol.

[13] Goetz M, Gradishar W, Anderson B (2019). NCCN guidelines insights: breast cancer, version 3.2018. J Natl Compr Cancer Netw.

[14] Paik S, Tang G, Shak S (2006). Gene expression and benefit of chemotherapy in women with node-negative, estrogen receptor-positive breast cancer. J Clin Oncol.

[15] Mamounas EP, Tang G, Fisher B (2010). Association between the 21-gene recurrence score assay and risk of locoregional recurrence in node-negative, estrogen receptor–positive breast cancer: results from NSABP B-14 and NSABP B-20. J Clin Oncol.

[16] Wolmark N, Mamounas EP, Baehner FL (2016). Prognostic impact of the combination of recurrence score and quantitative estrogen receptor expression (ESR1) on predicting late distant recurrence risk in estrogen receptor-positive breast cancer after 5 years of tamoxifen: results from NRG oncology/. J Clin Oncol.

[17] Dowsett M, Cuzick J, Wale C (2010). Prediction of risk of distant recurrence using the 21-gene recurrence score in node-negative and node-positive postmenopausal patients with breast cancer treated with anastrozole or tamoxifen: a TransATAC study. J Clin Oncol.

[18] Sparano JA, Gray RJ, Makower DF (2018). Adjuvant chemotherapy guided by a 21-gene expression assay in breast cancer. N Engl J Med.

[19] Mina L, Soule SE, Badve S (2007). Predicting response to primary chemotherapy: gene expression profiling of paraffin-embedded core biopsy tissue. Breast Cancer Res Treat.

[20] Esteva FJ, Sahin AA, Cristofanilli M (2005). Prognostic role of a multigene reverse transcriptase-PCR assay in patients with node-negative breast cancer not receiving adjuvant systemic therapy. Clin Cancer Res.

[21] Badve S, Baehner F, Gray R (2008). Estrogen- and progesterone-receptor status in ECOG 2197: comparison of immunohistochemistry by local and central laboratories and quantitative reverse transcription polymerase chain reaction by central laboratory. J Clin Oncol.

[22] Rhodes A, Jasani B (2009). The oestrogen receptor-negative/progesterone receptor-positive breast tumour: a biological entity or a technical artefact?. J Clin Pathol.

[23] Shen T, Brandwein-Gensler M, Hameed O (2015). Characterization of estrogen receptor-negative/progesterone receptor-positive breast cancer. Hum Pathol.

[24] Chan M, Chang MC, González R (2015). Outcomes of estrogen receptor negative and progesterone receptor positive breast cancer. PLoS One.

[25] Rakha EA, El-Sayed ME, Green AR (2007). Biologic and clinical characteristics of breast cancer with single hormone receptor positive phenotype. J Clin Oncol.

[26] Schroth W, Winter S, Bu¨ttner F (2016). Clinical outcome and global gene expression data support the existence of the estrogen receptor-negative/progesterone receptor-positive invasive breast cancer phenotype. Breast Cancer Res Treat.

[27] Fuqua SAW, Fitzgerald SD, Chamness GC (1991). Variant human breast tumour estrogen receptor with constitutive transcriptional activity. Cancer Res.

[28] De Maeyer L, Van Limbergen E, De Nys K (2008). Does estrogen receptor–negative/progesterone receptor–positive breast carcinoma exist?. J Clin Oncol.

[29] Hefti MM, Hu R, Knoblauch NW (2013). Estrogen receptor negative/progesterone receptor positive breast cancer is not a reproducible subtype. Breast Cancer Res.

[30] Allred DC (2008). Commentary: hormone receptor testing in breast cancer: a distress signal from Canada. Oncologist.

[31] Goldstein NS, Ferkowicz M, Odish E (2003). Minimum formalin fixation time for consistent estrogen receptor immunohistochemical staining of invasive breast carcinoma. Am J Clin Pathol.

[32] Qiu J, Kulkarni S, Chandrasekhar R (2010). Effect of delayed formalin fixation on estrogen and progesterone receptors in breast cancer: a study of three different clones. Am J Clin Pathol.

[33] Kunca M, Biernata W, Senkus-Konefka E (2018). Estrogen receptor-negative progesterone receptor-positive breast cancer – “Nobody’s land” or just an artifact?. Cancer Treat Rev.

[34] Onitilo A, Engel J, Greenlee R (2009). Breast cancer subtypes based on ER/PR and Her2 expression: comparison of clinicopathologic features and survival. Clin Med Res.

[35] Iwamoto T, Booser D, Valero V (2012). Estrogen receptor (ER) mRNA and ER-related gene expression in breast cancers that are 1% to 10% ER-positive by immunohistochemistry. J Clin Oncol.

[36] Allison KH, Hammond MEH, Dowsett M (2020). Estrogen and progesterone receptor testing in Breast cancer ASCO/CAP guideline update. J Clin Oncol.

